# Fetal Lymphoid Progenitors Become Restricted to B-1 Fates Coincident with IL-7Rα Expression

**DOI:** 10.1371/journal.pone.0165676

**Published:** 2016-10-28

**Authors:** Ryuji Iida, Kaori Shinoda, Yuki Hayano, Yoshinori Nagai, Kiyoshi Takatsu, Taku Kouro

**Affiliations:** 1 Laboratory of Immune Modulation, National Institutes of Biomedical Innovation, Health and Nutrition, Ibaraki, Osaka, Japan; 2 Department of Cell Cultures, National Institutes of Biomedical Innovation, Health and Nutrition, Ibaraki, Osaka, Japan; 3 Department of Immunobiology and Pharmacological Genetics, University of Toyama, Toyama-shi, Toyama, Japan; 4 JST, PRESTO, Kawaguchi, Saitama, Japan; 5 Toyama Prefectural Institute for Pharmaceutical Research, Imizu City, Toyama, Japan; B.C. Cancer Agency, CANADA

## Abstract

B-1 cells represent a sub-fraction of B lymphocytes that participate in T cell-independent antibody production and contribute to innate immunity. While the production of B-1 cells is favored during the fetal waves of lymphopoiesis, it has been unclear when and how that differentiation option is specified. To clarify this, lymphoid and hematopoietic progenitors of fetal liver (FL) and adult bone marrow (ABM) were examined for the B cell differentiation potential. Mouse common lymphoid progenitors (CLPs) and more primitive KSL fraction of FL and ABM were transferred to SCID mice and donor-derived B cell subsets were analyzed 4 weeks later. CLPs were also cultured on ST2 stromal cells for 6 days prior to transplantation. While Lin^-^ IL-7Rα^+^ CLPs from ABM differentiated to B-1, B-2 and marginal zone B (MZB) cells, equivalent cells from d15 FL differentiated mostly to B-1a cells. We found that fetal CLPs had less ability to colonize the bone marrow than adult CLPs. However, the fetal/adult difference was already present when progenitors were cultured in an identical condition before transplantation. More primitive KSL fraction of FL could generate the same broad spectrum of B cells typical of adults, including splenic MZB cells. In conclusion, we argue that FL and ABM-CLPs are intrinsically different regarding B-1/B-2 fates and the difference is acquired just before or coincident with the acquisition of IL-7Rα expression.

## Introduction

The humoral immune system is composed of functionally restricted lymphocyte subsets and some of them appear to make natural antibodies without deliberate immunization. B-1 cells are phenotypically distinguishable from conventional B-2 cells by their surface expression of CD43, CD5, IL-5Rα and absence of CD23 [1–3]. They also express CD11b in the peritoneal cavity but the expression is down-regulated in the spleen [4]. There is also “sister” population of B-1 cells that lack CD5, subdividing B-1 cells into CD5^+^ B-1a cells and CD5^-^ B-1b cells [5]. They can be activated in a T cell-independent manner immediately by microbial polysaccharides and self-antigens [6,7]. Thus B-1 cells are considered to represent the first line of defense against invading pathogens. B-1 cells have attracted considerable attention not only in that context but also because of their possible contribution to autoimmune diseases [8,9].

B-1 cells preferentially use certain immunoglobulin heavy chain genes and show skewed antigen specificity repertoires [10,11]. Therefore, it has been proposed that signals delivered via those receptors dictate B lineage fates [12]. This hypothesis was supported by the finding that most B cells in transgenic mice expressing a V_H_12 heavy chain transgene, representative of B-1 cell type B cell receptors (BCRs), were of the B-1 phenotype [13]. The importance of BCR signaling in B-1 cell development was also suggested by the phenotype of several mouse strains lacking signaling components of the B cell receptor, such as CD19, Vav or Btk, with few or no B-1 cells [14–16]. On the other hand, lineage marker negative (Lin^-^) CD93/AA4.1^+^ CD19^+^ CD45R/B220^Lo-Neg^ B-1 cell-specified progenitors have been isolated from fetal and adult mouse bone marrow [17,18]. These observations suggest B-1 determination can occur independently at BCR signaling. It seems possible that the B-1 cell formation can be favored at two levels, bias in early progenitors and selection of newly formed B cells on the basis of receptor specificity. The present study was designed to learn more about the initial branch point when progenitors are directed to B-1 cell fates.

Like other blood cells, lymphocytes are generated from hematopoietic stem cells (HSCs), through a process that involves gradual loss of differentiation options. Many stage-specific markers have been described, but fetal/adult differences have made it difficult to do side-by-side comparisons. Activation of the RAG1 locus corresponds to diminished myeloid potential and substantial restriction to lymphopoiesis, but early lymphoid progenitors identified on that basis in embryos still differ from those in adults [19,20]. There have been many definitions of common lymphoid progenitors in FL or ABM, but an expression of IL-7Rα has been consistently used [21–26]. Therefore, we isolated relatively large subsets according to IL-7Rα as one of the most widely used markers.

There is a drastic change in the progenitor potential of B-1 cells during ontogeny, that is active during fetal life vs. quite limited [17,18,27,28] or kept quiescent [29] in adults. The attenuation of B-1 cell development was accompanied by two models, a model based on an apparent wave of the HSC-independent progenitor which has B-1a committed potential [30] and another model based on selective loss of generating B-1a lineages in bona fide HSCs [31,32]. In the latter model, loss of B-1a potential gradually proceeds because many reports have shown the existence of B-1a long-lasting potential in the HSCs in neonatal and adult marrow [31,33,34]. Although these two concepts were not mutually elusive, a study by Ghosn et al challenged, demonstrating highly purified fetal as well as adult CD150^+^ CD48^-^ HSCs fail to generate B-1 cells [35]. Thus, it is still controversial whether fetal and adult CD150^+^ HSCs contribute to only B-2 cell differentiation or not. Therefore, one important goal of our study was to compare B-1 and B-2 differentiation potential of fetal and adult progenitors in the same stage.

Here we show that stem and primitive progenitor cells in the FL have the same broad differentiation potential as their adult counterparts. However, lineage progression in the fetal tissue is unique in imposing heavy bias towards B-1 lymphocyte fates. Expression of IL-7Rα was found to be a particularly informative milestone, inasmuch as it marked a point at which B-1/B-2 fate decisions were established.

## Materials and Methods

### Mice

BALB/cA and C.B-17/Icr-scid/scid (SCID) mice were purchased from CLEA Japan, Inc (Tokyo, Japan). Fetuses were obtained from pregnant mice at day 15 from vaginal plugs were observed (d15). All mice were housed and maintained under specific pathogen-free conditions in Research Animal Facility of National Institute of Biomedical Innovation. All animal experiments were planned in accordance with the guidelines of animal use at the National Institutes of Biomedical Innovation, Health and Nutrition and approved by an animal experiment committee of the Institutes with permission numbers DS19-88 and DS20-59.

### Antibodies

All antibodies used for FACS analysis were purchased from eBioscience (San Diego, CA) or BD Biosciences (San Jose, CA) unless otherwise noted.

### Preparation, staining and sorting of progenitors

Bone marrow cells were stained with purified anti-Gr-1, CD3, TER-119, B220, CD19 and CD11b mAbs and labeled cells were depleted with anti-rat IgG microbeads using LD columns with Quadro MACS magnet (Miltenyi Biotec, Gladbach, Germany). FL cells were depleted of erythroid cells using anti-TER-119 microbeads (Miltenyi Biotec). For detection of CLPs and KSL cells, cells were stained with FITC-lineage markers as well as PE-anti-Sca-1, AlexaFluor 647-anti-IL-7Rα, and PE-Cy7-anti-CD117/c-kit mAbs. The FITC-lineage marker cocktail consisted of following Abs; anti-IgM (Southern Biotech, Birmingham, AL), B220, CD19, Gr-1, DX5, TER-119, CD3e. Stainings with biotinylated antibodies were developed by streptavidin-PE. Stained cells were analyzed on LSR II flow cytometer (BD Bioscience), or purified on FACS Aria cell sorter (BD Bioscience). Flow cytometry data were analyzed with FlowJo software (Tree Star, Ashland, OR).

### Cell transfer experiment

Purified progenitors from d15 FL or 8-11wk ABM in 200 μL of PBS were intravenously injected into 6- to 9-week old SCID mice that were pre-treated with 1–2.5 Gy with an x-ray irradiation system through 0.2 mm copper + 0.5 mm aluminum filter (MBR-1520R-3; Hitachi Medical Corporation, Tokyo, Japan). At the indicated time point, mice were sacrificed and cells were collected from peritoneal cavities (PerC) and spleens (SPL). Cells were then counted, stained with FITC-anti-IgM, PE-CD23, PE-Cy7-CD5, APC-CD19, APC-eFluor780-CD45R and Pacific Blue-CD21 Abs and subjected to FACS analysis. In some experiments, cells were stained with FITC-CD24, PE-CD19, PE-Cy7-CD5, APC-CD93/AA4.1 and APC-eFluor780-CD45R.

### Intracellular staining

For detection of cytoplasmic μ chain (cμ), the cells were first stained with anti-IgM^a^ biotinylated mAb for surface μ chain (sμ), as well as PE-Cy7-B220 mAb and streptavidin-PE. Then, cells were fixed with 1% formaldehyde for 15min at room temperature and permeabilized with 0.2% Tween20 in staining buffer for 15min. Finally cμ was stained with FITC-conjugated F(ab’)_2_ fragment of goat anti-mouse IgM antibody (Southern Biotech).

### ELISA

For plate coating, L-α-phosphatidylcholine (PtC; Sigma Aldrich, St. Louis, MO) was dissolved in 70% ethanol at 30 μg/ml, inoculated in Maxisorp plates (Nunc, Rochester, NY) at 100 μl/well and the solvent was allowed to evaporate overnight at 37°C. Plates were blocked with 2% BSA in wash buffer (0.5 M NaCl, 10 mM MgSO_4_). After washing, samples were incubated and PtC-bound IgM was detected with goat anti-IgM biotinylated antibody (Southern Biotech) in combination with streptavidin-HRP (BD Bioscience). For measurement of serum IgM, plates were coated with rat anti-mouse IgM mAb (BD Bioscience) and PBS containing 0.05% tween 20 was used as wash buffer. For measurement of fecal IgA, fecal extracts were prepared from four pieces of fecal pellets in 800 μL of PBS supplemented with protease inhibitor cocktail (Complete, Mini; Roche, Mannheim, Germany) and subjected to ELISA with anti-IgA polyclonal antibody (Invitrogen, Carlsbad, CA) for capture in combination with HRP-anti-IgA polyclonal antibody (Southern Biotech) for detection. Each reaction was developed with o-phenylenediamine dihydrochloride (Sigma Aldrich) and H_2_O_2_ as a substrate and absorbance at 490 nm was measured.

### Cell cultures

CLPs were co-cultured for 6 days with 30Gy-irradiated ST2 stromal cells [36] in 24 well plate in RPMI 1640 medium supplemented with 5% FBS, 50 μM 2-ME, 100 U/ml penicillin and 0.1 mg/ml streptomycin. In some experiments, 1 ng/ml of recombinant mouse IL-7 (PeproTech Inc., Rocky Hill, NJ) was added to the medium. Non-adherent cultured cells were collected, counted and transferred to irradiated SCID mice or stained with FITC-anti-IgM, PE-Cy7-B220, APC-CD19 antibodies and Pacific Blue anti-I-A/I-E antibody that recognizes class II major histocompatibility complex (MHC II), for FACS analysis. Dead cells were excluded by 7-aminoactinomycin D (Sigma) staining.

### Statistical analysis

Statistical significances were calculated using Student’s single-tailed t-test. Shapiro-Wilk test was performed and the p values between fetal and adult marrow were more than 0.1.

## Results

### Lymphoid progenitors in the FL are heavily biased toward the B-1 lineage

**Fig 1:**
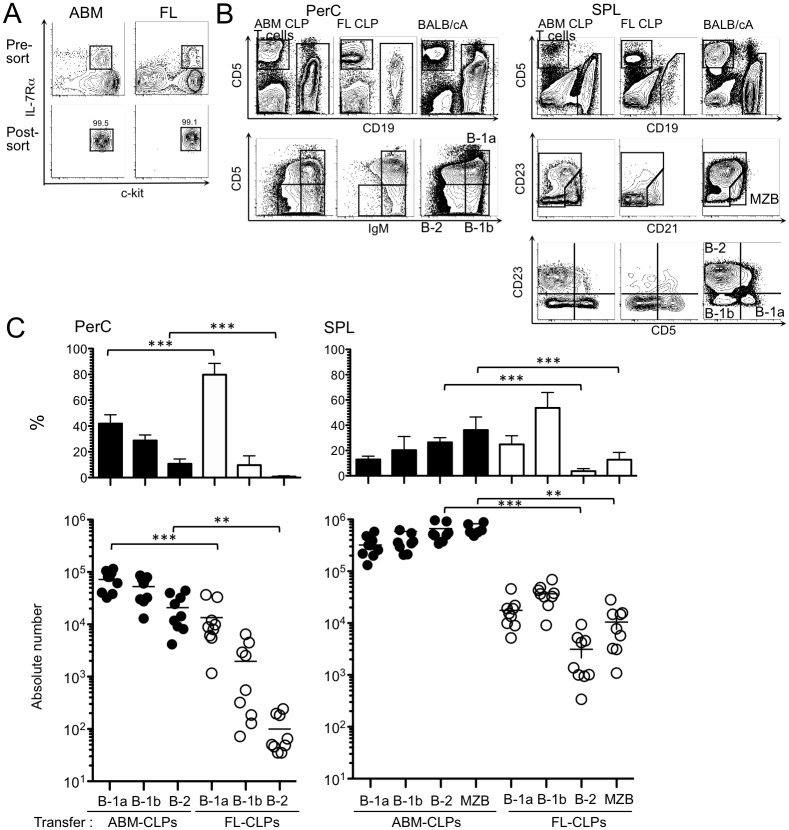
CLPs in the FL are heavily biased to produce B-1a cells. (A) CLPs were sorted according to the c-kit^+^ IL-7Rα^+^ cell gate from lineage-negative cell-enriched 8-wk-ABM and FL as indicated in the upper plots. Purities of CLPs were determined to be more than 99% by re-analysis (lower). (B) Representative staining profiles of the peritoneal cavity (PerC) and spleen cells (SPL) of SCID mice transplanted with 1.0 x 10^4^ cells of ABM-CLPs or FL-CLPs. Staining plots for a control BALB/cA mouse are also shown for reference. The upper panel shows gating for CD19^+^ B lineage cells, CD5^+^ CD19^-^ T lineage cells and the bottom panel shows CD19^+^ B cell subsets. MZB cells (CD19^+^ CD23^Lo-Neg^ CD5^-^ CD21^**Hi**^**)** were gated out from spleen CD19^+^ cell plots. Gating for B-1a cells (CD5^**+**^ CD23^-^), B-1b cells (CD5^-^ CD23^-^) and B-2 cells (CD5^-^ CD23^**+**^**)**. (C) Absolute numbers and percentages of B-1a, B-1b, B-2 and MZB cells in each mouse are shown. The data were pooled from three independent experiments (n = 9). (*P < 0.05, **P < 0.01, ***P < 0.001)

Recent reports indicated that proB cells in the adult and fetal bone marrow could be subdivided into B-1 and B-2 progenitors according to their expression of CD19 and CD45R/B220 [17]. On the other hand, FL proB cells were classically reported to generate only B-1 cells [27]. Since proB cells, including B-1 progenitors, are derived from CLPs [18,37], B cell fates of CLPs prepared from FL and ABM were directly compared by transplantation. There are many phenotypic descriptions of CLPs, and fetal ones may be unique in expressing, for example, CD11b. As a commonly used marker for both fetal and adult CLPs, IL-7Rα can be listed [20–22,38]. Consequently, we compared Lin^-^ c-kit^+^ IL-7Rα^+^ CLPs from d15 FL-CLPs to a similar population isolated from 8-wk- ABM-CLPs (Fig 1). ABM-CLPs (1.0 x 10^4^ cells/mouse) generated substantial numbers of spleen lymphocytes consisted of B-1a, B-1b and B-2 cells (Fig 1B and 1C). These categories of B cells were also abundant in the peritoneal cavity. In contrast, almost no CD19^+^ lymphocytes were recovered from spleens of mice transplanted with the same number of FL-CLPs, and nearly all of those in the peritoneum were B-1a cells (Fig 1B and 1C). Of note, CD21^Hi^ CD23^-^ marginal zone B cells (MZB) were seldom found in the spleens of SCID mice transplanted with FL-CLPs while they were abundant in recipients of ABM-CLPs (Fig 1C). Most of ABM-CLPs and FL-CLPs-derived CD19^+^ cells were IgM^+^ (Fig 1B), indicating they were mature B cells.

**Fig 2:**
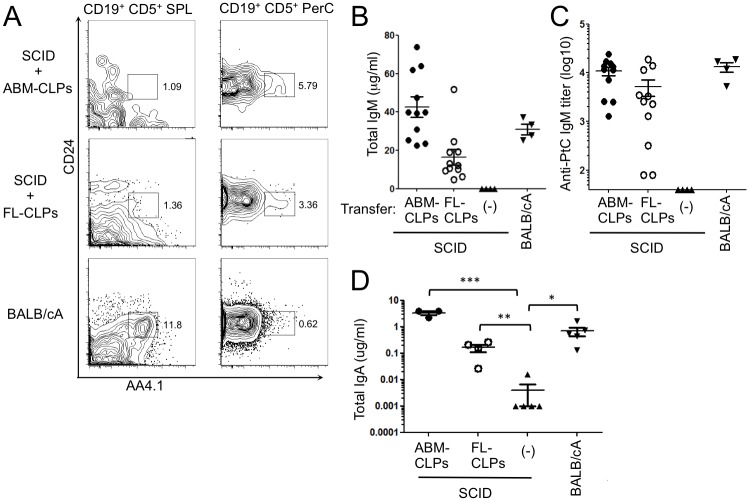
FL- and ABM-CLPs generate authentic B-1a cells and produce natural antibody reactive to phosphatidylcholine. SCID mice were reconstituted with 1 x 10^4^ CLPs from 8-wk-ABM or FL, and then anti-phosphatidylcholine Abs in serum were measured three weeks later. BALB/cA mice were used as controls. (A) PerC and SPL cells were stained with cell surface markers for transitional-1 (T-1) B cells as indicated. Plots are representative of 6 mice in each condition. (B) Total IgM concentrations and (C) phosphatidylcholine (PtC) specific IgM titers. Pooled from three experiments. (D) The concentration of IgA in the fecal extract. (*P < 0.05, **P < 0.01, ***P < 0.001)

The unique B-1a subset of lymphocytes shares some characteristics with newly formed, transitional (T1) cells, but the latter have the distinct high expression of CD24 and AA4.1 [39]. As shown in Fig 2A, CLPs-derived CD5^+^ B cells in the peritoneal cavity and spleen were AA4.1^-^ CD24^Lo^ and thus authentic B-1a cells. Substantial amounts of serum IgM were also made in these mice (Fig 2B) and, as is typical of B-1a cell-derived natural antibodies, showed reactivity to phosphatidylcholine, suggesting the production of functional B-1a cells (Fig 2C). The production of fecal IgA was also observed in the SCID mice transferred with either FL- as well as ABM-CLPs, showing generated B-1 cells reside and function also in the intestine (Fig 2D). We conclude that lymphoid progenitors in the FL are heavily biased to produce B-1a cells while those in the ABM have a full spectrum of B cell differentiation potential.

### Fetal progenitors do not efficiently engraft and differentiate in the bone marrow of recipient mice

**Fig 3:**
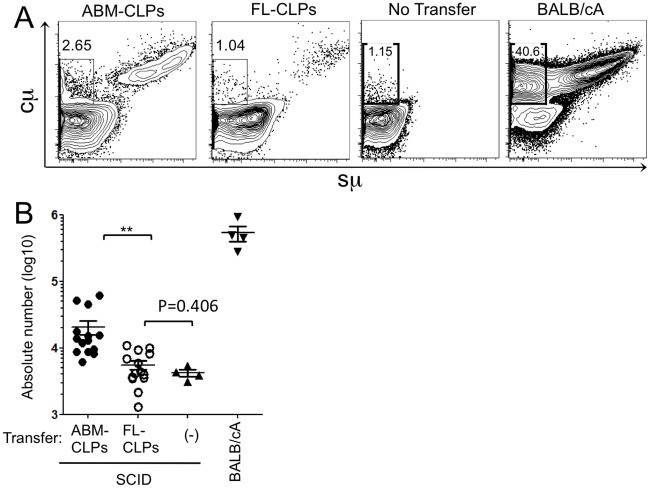
FL-CLPs do not efficiently engraft and differentiate in adult bone marrow. (A) Flow cytometry analyses are shown for bone marrow cells from SCID mice transplanted four weeks earlier with 8-wk-ABM- or FL-CLPs from BALB/cA mice. PreB cells (cμ^+^ sμ^-^) are indicated within the B220^+^ gate. (B) Absolute numbers of cμ^+^ sμ^-^ preB cells per femur. Each dot represents data form single mouse. The data are pooled from four experiments (n = 14). (**P < 0.01, N.S.: not significant)

Defective generation of the spleen and conventional B-2 cells by fetal progenitors might result from an inability to access normal environments that support B-2 lymphopoiesis. Due to dysfunction of Prkdc enzyme, SCID mice have defects in recombination of IgH gene and therefore they have few cytoplasmic μ chain (cμ) positive preB cells, and we used this as a means to measure donor-derived preB cells in the transplanted animals (Fig 3A). PreB cells were routinely detected in bone marrow of ABM-CLPs recipients, but the corresponding population in mice reconstituted with the same number of FL-CLPs were equivalent to non-transplanted background levels (Fig 3B). It is noteworthy that FL-CLPs-derived surface IgM positive cells are found in the bone marrow of FL-CLPs transferred mice, suggesting these cells had differentiated elsewhere and then circulated to the bone marrow (Fig 3A).

### Fetal lymphoid progenitors are functionally biased even when exposed to the same environment as their adult counterparts

**Fig 4:**
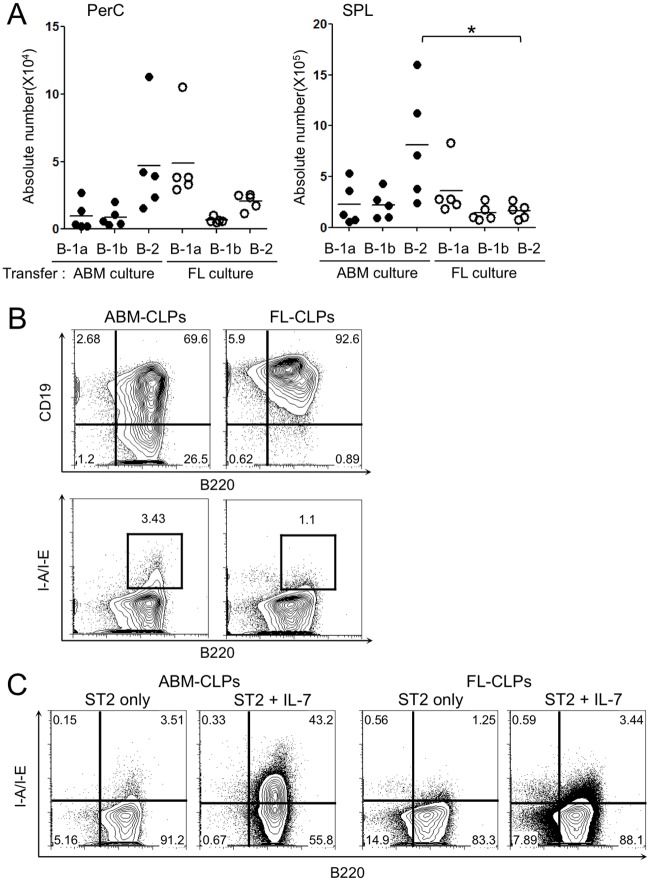
The B-1 biased fate of FL-CLPs is stable in culture. (A) Absolute numbers of B-1a, B-1b and B-2 cells in the SCID mice transferred with cells from cultures. FL-CLPs or 8-wk-ABM-CLPs were cultured on ST2 for 6 days and 4.0–5.0 x10^4^ cells were transferred to irradiated SCID mice. 3 weeks later, peritoneal and spleen cells were analyzed as described Fig 1B. (B) Surface markers of day6 ST2 cultures initiated with FL-CLPs or ABM-CLPs. The data are representative of two independent experiments with similar results (n = 5). (C) Expression of MHC II on the day6 ST2 cultures initiated with FL-CLPs or ABM-CLPs with or without IL-7. (*P < 0.05)

The inability of FL-CLPs to home to the bone marrow made us the reason that they might generate a diverse repertoire of B cells if exposed to an appropriate environment. Therefore, FL- and ABM-CLPs were sorted and cultured for 6 days on monolayers of the bone marrow derived ST2 stromal cell line, which produce IL-7 and support early B lymphopoiesis [40]. Differentiation potential was then tested by transplanting 4.0–5.0 x10^4^ cells of the two types of cultured cells to immunodeficient mice. FL-CLPs derived cells preferentially generated B-1a cells, while ones from the ABM-CLPs cultures produced a majority of B-2 cells (Fig 4A). Supporting this, cultures initiated with ABM-CLPs mainly consisted of CD19^+^ B220^+^ and CD19^-^ B220^+^ cells and in contrast, most of the cells in FL-CLPs cultures were CD19^+^ B220^+^ or CD19^+^ B220^-^ cells. Further, ABM-CLPs-derived culture contained slightly, but constantly the high number of class II major histocompatibility complex (MHC II) expressing cells than FL-CLPs-derived culture (Fig 4B). The difference in the number of MHC II expressing cell became much more obvious when recombinant IL-7 was further added to the culture (Fig 4C). The differential behaviors of FL- and ABM-CLPs in the identical condition indicate that B-1 versus B-2 decision is determined prior to the CLPs stage.

### B lineage fates are determined in primitive lymphopoietic cells

**Fig 5:**
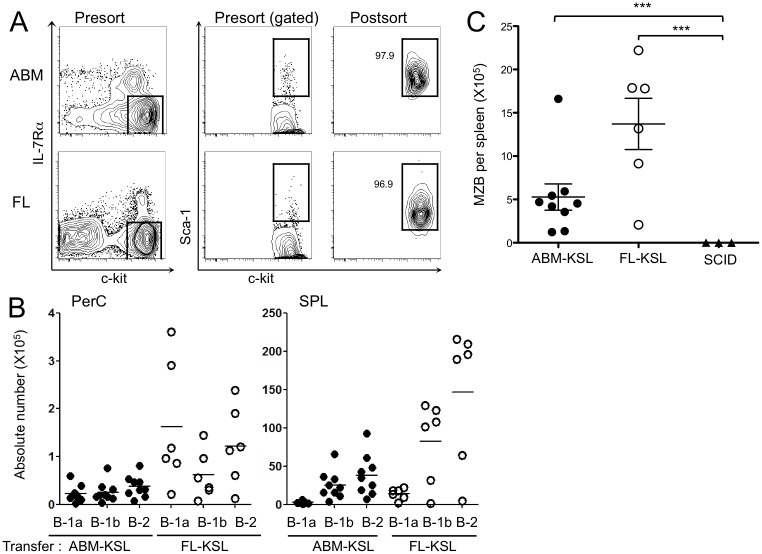
Primitive hematopoietic cells in the FL are uncommitted and can generate all B lymphocyte subsets. (A) Sorting gate (left and center) and post-sort purity analysis (right) for c-kit^Hi^ IL-7Rα^-^ Sca-1^+^ KSL cells from 8-wk-ABM or FL are shown. (B) Absolute numbers of B-1a, B-1b and B-2 cells in PerC and SPL as well as (C) MZB cells in the SPL of SCID mice after 4 weeks from injection of 1.0 x 10^3^ FL- or ABM-KSL cells. Data from three independent experiments were pooled. (***P < 0.001)

In contrast to our findings, other investigators have found that fetal hematopoietic cells can generate all categories of B lymphocytes [28]. A possible explanation might lie in nature of cells that were transplanted. In fact, when the larger population of FL cells, that is whole FL just depleted of TER119^+^ cells by MACS, could generate both B-1 and B-2 cells in the SCID mice (data not shown). The Lin^-^ Sca-1^+^ c-kit^Hi^ (KSL) cell fraction of marrow and FL is enriched for stem and progenitor cells more primitive than CLPs (Fig 5A). We transplanted 1.0 x 10^4^ cells of these subsets to SCID mice and evaluated differentiation potential 4 weeks later. In contrast to results obtained from transplants of FL-CLPs (Fig 1), in addition to PerC B-1a cells, there were significant numbers of B-1b and B-2 cells in the PerC and spleen in mice transplanted with FL-KSL cells resembling ones transplanted with ABM-KSL cells (Fig 5B). Furthermore, the KSL fraction of the FL was able to generate marginal zone B cells (Fig 5C). Thus, stem and primitive progenitor cells in the FL have the potential to generate all categories of B lineage lymphocytes, indicating options are dramatically reduced at or before the CLP stage.

## Discussion

B-1 lymphocytes appear to be highly specialized and may have unique roles in host defense. However, it has long been debated if they arise only during fetal life and if the specificity of their antigen receptors drives their formation. A key question has been when lymphoid progenitors become committed to B-1 versus conventional B-2 cell fates and if this choice is irrevocable. We have now used an immunodeficient mouse reconstitution model to learn that primitive stem and progenitor cells in FL or ABM can make the full spectrum of B lineage lymphocytes. However, fetal progenitors at the CLP stage are heavily biased to produce B-1a cells that colonize the peritoneal cavity. This property is paralleled by unique homing properties, but that did not entirely account for fetal/adult differences. Fetal progenitors were B-1 biased even when differentiated in lymphopoietic cultures and transplanted. In contrast to FL-CLPs, those present in adult marrow can replenish all lymphocyte subsets. Thus, we concluded B lineage options are defined early in the hematopoiesis, before or upon acquisition of IL-7Rα.

ABM was long believed to lack B-1 cell progenitors or to be restricted to the production of the B-1b subset, if any [28]. However, consistent with recent reports [18,33,34], we observed the B-1a cell formation from transplanted ABM-CLP. We confirmed that they were distinct from newly formed tansitional-1 B cells and produced the phosphatidylcholine reactive IgM that is the hallmark of B-1a cells. Bone marrow may produce small numbers of B-1 cells relative to conventional B cells in steady state adult mice, but the potential is certainly there.

Recent studies report neonatal bone marrow CLPs to be more efficient in making B-1 cells than ABM-CLPs but both can make a significant number of B-2 cells [31,32]. In sharp contrast, our data show that FL-CLPs makes few B-2 cells (Fig 1). It is possible that in addition to the gradual loss of B-1 potential from neonatal to adult animal, there is an emergence of B-2 potential between d15 fetus and neonate. Alternatively, our findings don’t conflict with an embryonic wave of B-1a lymphopoiesis prior to the emergence of definitive HSCs [30]. Although we cannot rule out that these progenitors become FL- CLPs or neonatal bone marrow CLPs, we observed B-1a generation in ABM- CLPs. What we have demonstrated is not that the descendants from HSC-independent embryonic wave become FL-, neonatal- and ABM- CLPs but the potential for making B-1a cells is there. The difference of B-2 potential may be explained by the difference of hematopoietic organs, that is liver versus bone marrow. In fact, surveillance of developing preB cells of donor origins, we found FL-CLPs do not differentiate in the bone marrow (Fig 3). Although CXCR4 is known to be required for homing and retention of lymphoid progenitors in bone marrow [41,42], we found expression of CXCR4 on FL-CLPs comparable to ABM-CLPs (unpublished observation). Since we have no means to force-migrate FL-CLPs to bone marrow, we cannot directly observe the effect of bone marrow environment to B-2 differentiation. Instead, culturing FL-CLPs on the bone marrow-derived stromal cell line did not induce the B-2 potential while same culture condition rather enhanced B-2 skewing of ABM-CLPs (Fig 4), suggesting FL-CLPs are intrinsically and B-1-committed.

The more primitive “KSL” fraction of FL hematopoietic progenitors showed flexibly in B-1/B-2 fate decision. In addition to reports by Barber et al. [31] using transplantation setting, a single cellular barcoding technique showed B-1/B-2 bi-potential of FL LT-HSCs [32]. The genetic transition in HSCs during ontogeny resulted in the diminished potential to generate B-1a cells in single cell levels [31]. Our findings are consistent with those reports, but are in sharp contrast with a model by Ghosn in which B-2 lineage restriction is irrevocably established at the CD150^+^ CD48^-^ KSL level and only the pathway toward B-1a lymphopoiesis is the derivative of the embryonic wave of HSC-independent lymphopoiesis during ontogeny [35]. The genetic changes in HSCs should be affected by epigenetic modifiers / environmental factors / mouse strains. Indeed, transduction of fetal epigenetic modifiers, Lin28b into ABM KSL cells leads to the potential changes to give rise to B-1/B-2 cells. FL-KSL cells should differentiate to B-1-committed CLPs when kept in the FL because CLPs in the FL are all committed to B-1 lineage. In contrast, our transfer experiments suggest FL-KSL cells can also make B-2 committed CLPs when placed in the adult environment. It is noteworthy that upon transfer to SCID mice FL-KSL cells showed more efficient B cell production than ABM-KSL cells, but the B-1/B-2 ratio was essentially the same. Together with the fact FL-CLPs make few B-2 cells, both *in vivo* and *in vitro*, we argue that KSL cells are not committed to either B-1 or B-2 lineage and fate decision depends on the environment during differentiation to CLP stage. To confirm this, the further trial such as injection of ABM-KSL cells into fetuses in the uterus would be needed.

Although we have not obtained concrete evidence for mechanistic differences that explain intrinsic differences in the B-1/B-2 fates between FL- and ABM-CLPs, one superficial difference is that MHC II positive cells are generated only from ABM-CLPs under identical culture condition. MHC II is expressed on proB cells in the ABM but not on those in the FL [43]. Even in the ABM, MHC II is not expressed on CLPs yet (data not shown and [30]). Therefore, it might be suggested that FL-CLPs and ABM-CLPs are different in the regulation of MHC II expression. It is noteworthy that the addition of IL-7 to these cultures drastically enhanced MHC II molecules on ABM-CLPs but not on FL-CLPs (Fig 4C). This is the direct effect of IL-7 to CLPs but not an indirect effect via ST2 stromal because the same result was obtained with the stroma-free culture containing stem cell factor and Flt3-ligand with or without IL-7 (data not shown). This fact indicates not only that induction of MHC-II is dependent on IL-7R signaling, but also that there is a qualitative difference in the IL-7R signaling between FL- and ABM-CLPs. Since both FL- and ABM-CLPs equally express IL-7Rα and proliferate in response to IL-7, it is likely that there is no difference at least in the IL-7-induced proliferation signaling between FL- and ABM-CLPs. Differences in the instructive signaling pathway of IL-7R including epigenetic regulation of certain target gene(s) should exist between B-1-biased and B-2-biased CLPs. Although further inquiries for such factors and genes will be needed to elucidate mechanisms for B-1/B-2 fate decision, current data suggest IL-7R signaling as an important candidate.

Our observation of B-1/B-2 bipotent differentiation of ABM-CLPs raised another question, that is whether each ABM-CLPs are B-1/B-2 bipotent or they are the mixture of committed B-1 and B-2 progenitors. Clonal analysis in the recent report suggests latter because there was no B-1/B-2 double positive well [31]. Our finding of pure B-1 potential in the FL-CLPs also supports this hypothesis. Comparing gene expression of FL-CLPs to ABM-CLPs by cDNA subtraction or microarray analysis will be the promising way to search for yet to be identified B-1 lineage marker at this earliest stage.

Since in contrast to FL- and ABM KSL cells, CLPs generated quite limited numbers of B-1b cells (Figs 1 and 4), above-mentioned discussions about B-1 cell commitment will be applied to CD5^+^ B-1a cells. The ratio of B-1a cells and B-1b cells seems to depend largely on mouse strain (data not shown) and extent of B-1b cell reconstitution also differ among research groups and protocols such as recipient mice, the source of progenitors etc. [18,29]. Recent findings indicate B-1b cells are also functionally different from B-1a cells regarding their contribution to thymus-independent antigen-specific antibody production and the ability to form immunological memory [44]. Thus, it is possible that B-1b cell differentiation is regulated in a different manner from B-1a cells.

Contrary to B-1 lineage cells, the formation of MZB cells was compromised in the fetal CLP transplantation. Both MZB and B-1 cells are considered to be components of the innate, T cell-independent humoral immune system. Recently, lymphoid progenitors in the d9 yolk sac and intra-embryonic hemogenic endothelium were reported to differentiate into both B-1 cells and MZB, but not to B-2 cells [31]. However, the existence of independent MZB progenitor is also suggested because MZB is produced only one-third of recipient mice transferred with B-1 progenitors from adult spleen [29]. Our observation of the complete absence of MZB potential in the FL-CLPs supports the latter hypothesis that MZB progenitor is different from B-1 lineage progenitors but just indistinguishable in the stages of ontogeny except for d15 FL at least.

## Conclusions

Our new findings address a number of controversies and show that the fetal wave of B lymphopoiesis is shunted into the B-1a lineage prior to, or coincident with, the acquisition of IL-7Rα, which is the distinguishing characteristic in all definitions of CLPs. Beyond that point, the progenitors are intrinsically prone to generate this functionally unique component of the humoral immune system. The potential is retained in adults, though the display of IL-7Rα on progenitors no longer denotes B-1a restriction.
